# MRI-Based Multiscale Model for Electromagnetic Analysis in the Human Head with Implanted DBS

**DOI:** 10.1155/2013/694171

**Published:** 2013-07-15

**Authors:** Maria Ida Iacono, Nikos Makris, Luca Mainardi, Leonardo M. Angelone, Giorgio Bonmassar

**Affiliations:** ^1^Athinoula A. Martinos Center For Biomedical Imaging, Department of Radiology, Massachusetts General Hospital, Harvard Medical School, Charlestown, MA 02129, USA; ^2^Division of Physics, Office of Science and Engineering Laboratories, Center for Devices and Radiological Health, US Food and Drug Administration, Silver Spring, MD 20993, USA; ^3^Center for Morphometric Analysis, Department of Psychiatry and Neurology, Massachusetts General Hospital, Harvard Medical School, Boston, MA 02129, USA; ^4^Bioengineering Department, Politecnico di Milano, 20133 Milan, Italy

## Abstract

Deep brain stimulation (DBS) is an established procedure for the treatment of movement and affective disorders. Patients with DBS may benefit from magnetic resonance imaging (MRI) to evaluate injuries or comorbidities. However, the MRI radio-frequency (RF) energy may cause excessive tissue heating particularly near the electrode. This paper studies how the accuracy of numerical modeling of the RF field inside a DBS patient varies with spatial resolution and corresponding anatomical detail of the volume surrounding the electrodes. A multiscale model (MS) was created by an atlas-based segmentation using a 1 mm^3^ head model (mRes) refined in the basal ganglia by a 200 **μ**m^2^ ex-vivo dataset. Four DBS electrodes targeting the left globus pallidus internus were modeled. Electromagnetic simulations at 128 MHz showed that the peak of the electric field of the MS doubled (18.7 kV/m versus 9.33 kV/m) and shifted 6.4 mm compared to the mRes model. Additionally, the MS had a sixfold increase over the mRes model in peak-specific absorption rate (SAR of 43.9 kW/kg versus 7 kW/kg). The results suggest that submillimetric resolution and improved anatomical detail in the model may increase the accuracy of computed electric field and local SAR around the tip of the implant.

## 1. Introduction

The use of magnetic resonance imaging (MRI) as a diagnostic tool is increasing, with approximately 30 million scans performed in United States in 2007. Approximately 300,000 patients per year implanted with active implanted medical devices, such as pacemakers, deep brain stimulators (DBSs), interventional guidewires, and cochlear implants are denied MRI because of safety concerns [1], including radiofrequency- (RF-) induced heating of the tissues near the implanted device [2–5]. When patients with conductive implanted devices undergo MRI, the RF field used to elicit the signal is picked by the conductive lead (antenna effect) inducing currents along the lead that flow into the surrounding tissues [6, 7]. Such currents may induce high levels of energy—and related possible thermal damage of the tissue—localized in a small volume surrounding the distal tip of the implant [8–11]. Serious injuries related to RF-induced heating have been reported in two patients with DBS: the first experienced a temporary dystonia [12] and the second suffered a permanent hemiparalysis [13]. The maximum allowable RF energy absorbed by the patients during MRI is limited by the Food and Drug Administration (FDA) and International Electrotechnical Commission (IEC) guidelines which specify the levels of specific absorption rate (SAR) averaged over the whole body, whole head, and the peak local SAR averaged over any 1 g and 10 g of tissue [14, 15]. The SAR in patients with implanted devices undergoing MRI has been widely studied in the literature using numerical methods [9, 11, 16, 17]. These computational studies were performed using coarse (≥1 mm^3^) uniform geometrical meshes for the modeling of the human anatomy and the associated implants [11, 16, 18, 19]. However, accurate estimation of the electric fields near thin (≪1 mm) metallic wires is difficult unless a sub-millimetric mesh is used to discretize the geometry of the implant, as suggested by [9]. Elwassif and colleagues [20] recently reported that increased precision in modeling a DBS electrode allowed for improved accuracy of the computed RF heating. However, the model did not include specific anatomical information of the structures surrounding the electrode. 

The goal of this study was to explore whether a more detailed model of the tissue surrounding the implant could affect the computation of the RF-induced electric field surrounding the electrode, where the highest and potentially harmful values of electric field are expected. For this purpose, we designed a multi-scale bioelectromagnetic model of a human head with an implanted DBS positioned in the globus pallidus internus (GPi). The procedure was composed of the following steps: (i) generation of the multi-scale anatomical model of the head, (ii) electrical modeling of the tissues and the electrode, and (iii) computation and comparison of the electric field and SAR in the uniform and multi-scale configurations. The multi-scale model was generated from a 1 mm^3^ MRI-based whole-head model [11, 21]. While the 1 mm^3^ spatial resolution of the existing model was adequate to precisely outline several anatomical structures, it was not sufficient to accurately resolve the DBS electrode, the DBS target (i.e., the GPi), and the deep brain anatomical structures in its vicinity. The major challenge was then improving the anatomical detail of the existing 1 mm^3^ model in the volume surrounding the electrode. For this purpose, we exploited the information derived from a 7 Tesla 200 *μ*m^3^ ex vivo brain dataset allowing identification of many anatomical details not observable using 1.5 or even 3 T MRI [22, 23]. The micro-resolution dataset was aligned to the milli-resolution model using a non-rigid registration and used as atlas to segment and outline the major basal ganglia nuclei on the latter. Multi-scale modeling with both milli- and micro-metric resolutions was used in order to calculate in a reasonable computing time (i.e., about three days) a precise solution of the electric field and SAR generated by an MRI head coil at 128 MHz over the entire head. We then compared the results obtained using the multi-scale model with those of the original 1 mm^3^ uniform head model used in [11, 24] in order to assess how the resolution affected the electric solution. 

## 2. Materials and Methods

### 2.1. Geometric Model

#### 2.1.1. Anatomical Head Model

**Figure 1:**
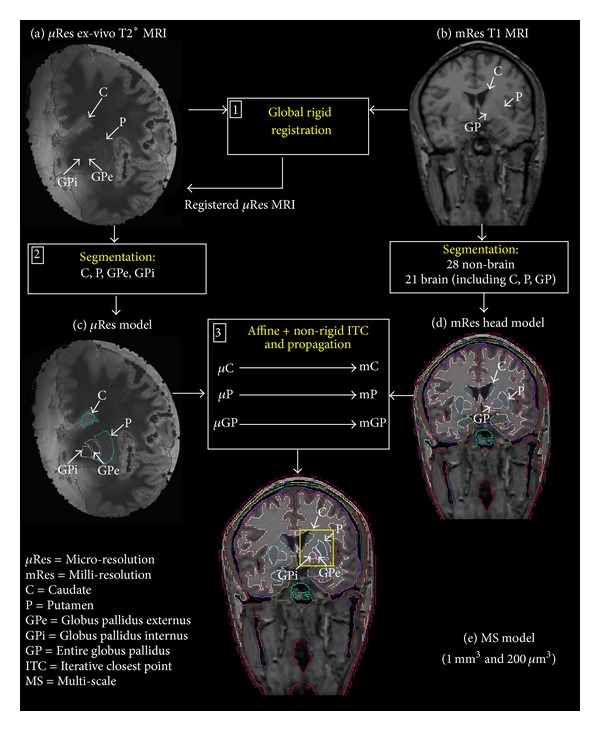
Workflow of the procedure for multi-scale (MS) model generation (e). Step 1: the original MRI datasets (a) *μ*Res and (b) mRes were first rigidly registered. Step 2: the contours of the target nucleus (i.e., GPi) and the surrounding major basal ganglia nuclei were segmented on the registered *μ*Res dataset to generate the *μ*Res model (c). Segmentation and generation of the mRes head model (d) were described in [21]. Step 3: each segmented structure in the *μ*Res model—that is, the caudate (*μ*C), the putamen (*μ*P), and the globus pallidus (*μ*GP, i.e., combined *μ*GPe and *μ*GPi)—was registered with its corresponding structure in the mRes head model (mC, mP, and mGP, resp.) using a non-rigid version of the iterative closest point (ICP). The *μ*Res model structures were propagated on the mRes model (label propagation) and the resulting dataset was a multi-scale (MS) model (e) enhanced in the basal ganglia (yellow square).

An ex vivo anatomical dataset was acquired and integrated with an existing MRI-based dataset of the head to obtain the final model.  Figure 1 shows the workflow of the procedure used to generate the multi-scale (MS) head model. The data were as follows: (i) a milli-resolution (mRes) head model previously implemented in [21]; (ii) an ex vivo micro-resolution (*μ*Res) model (i.e., the segmented postmortem brain hemisphere at 200 mm^3^); and (iii) the MS head model resulting from the integration of the *μ*Res and the mRes head models.

*The Millimetric Resolution (mRes) Head Model.* The preexisting head model was generated as described in [21] using a 1 mm^3^ resolution T1-weighted MRI of a healthy adult male (Figure 1(b)). In this dataset, 28 nonbrain and 21 brain structural entities were distinguished and segmented, including the caudate (C), the putamen (P), and the globus pallidus (GP), which was segmented as a unique structure without discerning between its internal and external parts (Figure 1(d)).

*Ex Vivo Microresolution (*μ*Res) Model*. A healthy brain postmortem hemisphere was selected for the construction of the *μ*Res MRI-based model.  Figure 1(a) shows the T2* MRI dataset that was acquired as follows: 200 *μ*m^3^ isotropic resolution, TR/TE/flip = 40 ms/20 ms/20°, and 1600 × 1100 × 896 matrix [25]. The contours of the target nucleus (i.e., GPi) and the surrounding major basal ganglia nuclei (i.e., caudate, putamen, and the external part of the globus pallidus, i.e., GPe) were manually outlined on the *μ*Res dataset (Figure 1, step 2). The caudate segmentation included the head, the body, and the tail of the nucleus caudatus. The nucleus accumbens was included in the caudate segmentation as it is ontogenetically and phylogenetically related to the caudate. Putamen and globus pallidus (GP), which lie alongside to form a lens-shaped nucleus, were separated using the external medullary lamina—a thin layer of white matter dividing the two nuclei and visible on the images. In the same way, the internal medullary lamina was used as a landmark to divide the GP in its external and internal parts and thus for outlining the GPe and the GPi. The result of the segmentation was a label mask, namely, the *μ*Res model (Figure 1(c)).

*Multi-Scale (MS) Head Model.* The mRes head model was used as a starting reference model. The *μ*Res model was used as atlas and registered on the mRes head model in order to outline the target, the GPi, where the electrode was positioned, and to refine the structures that surrounded the target, namely, the caudate, the putamen, and the GPe (atlas-based segmentation). The registration of the *μ*Res model (floating dataset) on the mRes head model (reference dataset) was performed by a preliminary global landmark-based registration (Figure 1, step 1), followed by a local surface-based registration (Figure 1, step 3). For the preliminary landmark-based rigid registration (Figure 1, step 1), each MRI volume of the two models was used to manually identify a set of three noncollinear corresponding landmarks: the anterior commissure (AC), the posterior commissure (PC), and the superior point of the interhemispheric fissure. Orientation and position of the datasets were corrected by aligning the centroids of the two sets of points and then the floating image was rotated and scaled by minimizing the sum of the squared displacements between the three corresponding points in the two volumes. 

**Figure 2:**
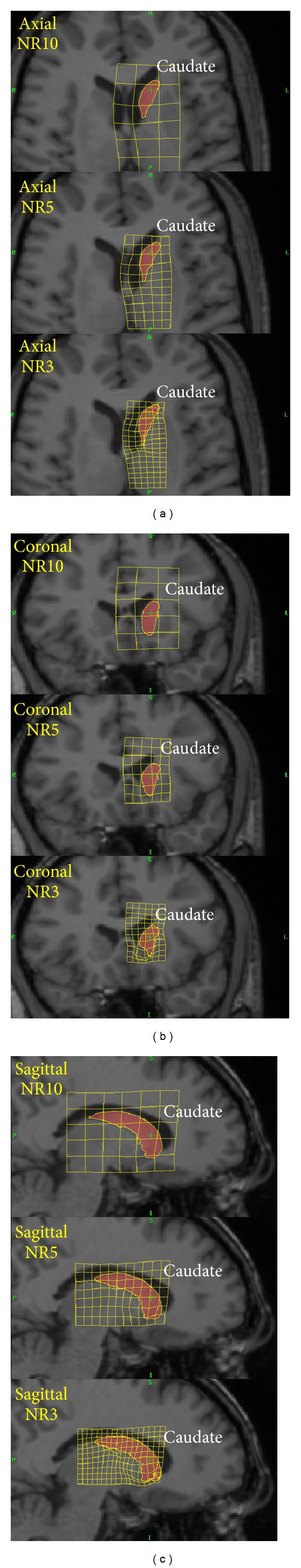
Axial (a), coronal (b), and sagittal (c) views of the deformed grids at three different resolution levels—that is, control point spacing of 10 mm^3^ (NR10) (top row), 5 mm^3^ (NR5) (middle row), and 3 mm^3^ (NR3) (bottom row)—resulting from the caudate coarse-to-fine non-rigid (NR) registration.

After the rigid registration, we generated corresponding meshes by directly triangulating the homologous structures segmented on both the floating and the reference datasets. Then, each mesh of points—parameterizing the caudate, the putamen, and the globus pallidus (GP, i.e., combined GPe and GPi)—in the *μ*Res model was registered with the corresponding mesh in the mRes head model using a structure-specific surface-based registration procedure based on a non-rigid version of the iterative closest point (ICP) algorithm [26]. The non-rigid registration was applied in a coarse-to-fine fashion by manipulating underlying free form deformation (FFD) meshes of control points with increasing resolution. At each mesh resolution level *L*, a continuous and smooth deformation field was obtained by interpolating the control points using a set of B-spline basis functions [27, 28]. Let Φ^*L*^ denote a *n*_*x*_ × *n*_*y*_ × *n*_*z*_ mesh of control points Φ_*i*,*j*,*k*_^*L*^ corresponding to the level *L* and uniformly spaced of *δ*. Then, the FFD can be written as the 3D tensor product of the familiar 1D cubic B-splines:

(1) $$\begin{array}{c} T^{L}\left(x,y,z\right)=\overset{3}{\underset{l=0}{\sum }}\overset{3}{\underset{m=0}{\sum }}\overset{3}{\underset{n=0}{\sum }}B_{l}\left(u\right)B_{m}\left(v\right)B_{n}\left(w\right)\Phi_{i+l,j+m,\;k+n}^{L} \end{array}$$

where *i* = ⌊*x*/*n*_*x*_⌋ − 1, *j* = ⌊*y*/*n*_*y*_⌋ − 1, *k* = ⌊*z*/*n*_*z*_⌋ − 1, *u* = *x*/*n*_*x*_ − ⌊*x*/*n*_*x*_⌋, *v* = *y*/*n*_*y*_ − ⌊*y*/*n*_*y*_⌋, *w* = *z*/*n*_*z*_ − ⌊*z*/*n*_*z*_⌋, and *B*_*l*_ represents *l*th the basis function of the B-spline [29]. Furthermore, the deformation field of the level *l* initializes the deformation field of level *l* + 1 following a hierarchical scheme. This allowed for a gradual adjustment of the corresponding meshes while increasing the level of detail of the registration. We used for registration six different resolution levels with FFDs control point spacing of 20 mm^3^, 10 mm^3^, 5 mm^3^, 3 mm^3^, 1 mm^3^, and 0.5 mm^3^, respectively.  Figure 2 shows three grids at different resolutions used in the hierarchical coarse-to-fine caudate registration. The accuracy of each structure-specific transformation was measured at each resolution level by the root mean square error (RMSE) between the corresponding points of the two meshes under registration.

*Label Propagation. *For each resolution level and for each structure, we benchmarked the accuracy of the matching of the structures identified by the registration procedures with those originally delineated by two anatomists in agreement (i.e., the ground truth or GT) with the mRes head model by evaluating several metrics. The metrics were the following:

- the distance between the centroids (Dc) of the algorithmically and the manually outlined structures,
- the percent match, PM = [TP/GT] · 100 = [TP/(TP + FN)] · 100,
- the positive predictive value, *P* + = [TP/(TP + FP)] · 100,

where for each structure, TP = true positives, that is, pixels labeled as belonging to the structure in the GT and by the algorithm; FP = false positives, that is, pixels labeled as belonging to the structure but not within GT; and FN = false negatives, that is, pixels falsely marked as background. For both PM and *P*+, an ideal value is 100%, when the algorithm perfectly localizes the structure's pixels.

Once the *μ*Res structures in the atlas fitted the corresponding mRes structures of the head model, the estimated structure-specific transformations were used to propagate onto the mRes head model (label propagation; Figure 1, step 3) the details of the caudate, putamen, and GP outlined on the *μ*Res dataset.  Figure 1(e) shows the integration of the two datasets, which results in the enhancement of the mRes head model in a 3.2 × 6.28 × 3.36 cm^3^ volume containing the basal ganglia (yellow square). The generated dataset is a multi-scale (MS) model with both milli- and micro-metric resolutions.

#### 2.1.2. Deep Brain Stimulation Implant Model

**Figure 3:**
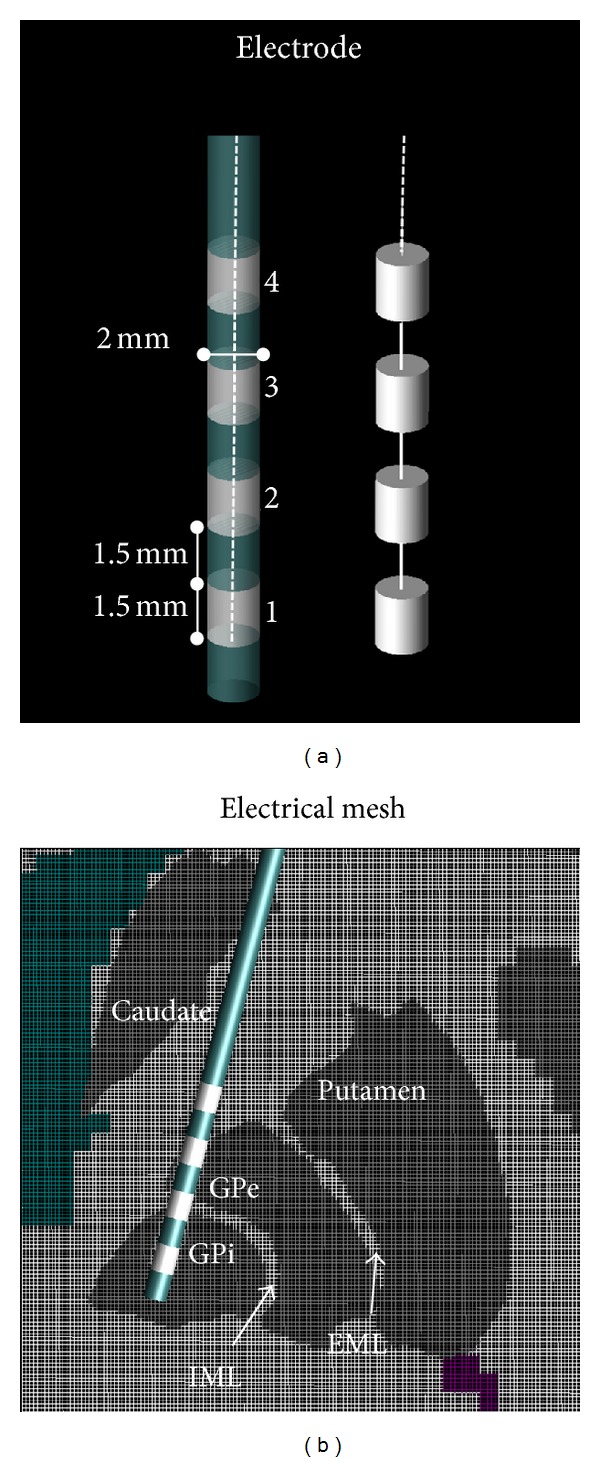
Electrical model: (a) model of the electrodes and (b) a zoomed view of the microresolution mesh around the electrode. Caudate, putamen, GPe, and GPi were labeled as grey matter (*σ* = 0.58 S/m, *ε*_*r*_ = 73.51). The DBS is made of platinum/iridium conductor wire and electrodes (*σ* = 4 · 10^6^ S/m) with 80 A urethane insulation (*σ* = 10^−10^ S/m, *ε*_*r*_ = 3) [20]. The high resolution of the model allowed to outline and precisely characterize both geometrically and electrically small anatomical details such as the white matter (*σ* = 0.34 S/m, *ε*_*r*_ = 52.53) between the GPe and the GPi (i.e., the internal medullary lamina IML) and between the GPe and the putamen (i.e., the external medullary lamina EML) and the caudoputaminal bridges (grey matter).

One left unilateral DBS implant was modeled for the study using AutoCAD (Autodesk, Inc., CA). The configuration of the lead was based on [11] with the following specific changes implemented: (i) the distal part of the lead was moved to target at the left GPi, (ii) the wire was created as a smoothed and continuous cubic spline passing through the extremities of the 19 segments described in [11] and the insulation was generated by sweeping a 1 mm radius circle along the spline, and (iii) the distal end of the implant was modeled as an insulated lead with an array of four cylindrical electrode contacts following the design of a commercially available deep brain stimulator [20], (Figure 3(a)). The four electrodes were connected by the conducting wire as shown in Figure 3(a). 

### 2.2. Electrical Model

The anatomical model was converted into a bioelectromagnetic model by assigning to each anatomical structure the respective conductivity and permittivity at 128 MHz [11, 30]. Following the anatomical definition of basal ganglia [31], the nuclei segmented using the *μ*Res atlas were assigned the electrical properties of grey matter (*σ* = 0.58 S/m, *ε*_*r*_ = 73.51) [11, 32] embedded in white matter (*σ* = 0.34 S/m, *ε*_*r*_ = 52.53) of each cerebral hemisphere and adjacent to CSF in the ventricular space. As an improvement of the mRes model [11], the micro-resolution model allowed to outline and precisely characterize both geometrically and electrically small anatomical details such as the white matter between the GPe and the GPi (i.e., the internal medullary lamina IML), and between the GPe and the putamen (i.e., the external medullary lamina EML) and the caudo-putaminal bridges (grey matter).  Figure 3 shows the electrode geometry (a) and a magnified view of the micro-resolution electric mesh around the electrode in the MS model (b). The electrical parameters were considered to be linear with electric field, nondispersive, isotropic, and heterogeneous in space. The DBS is made of platinum/iridium conductor wire and electrodes (*σ* = 4 · 10^6^ S/m) with 80 A urethane insulation (*σ* = 10^−10^ S/m, *ε*_*r*_ = 3) [20, 33]. 

#### 2.2.1. Finite-Difference-Time-Domain Simulations

The MS model was tested by calculating the electric field generated by an RF birdcage coil [11] at 128 MHz (i.e., approximate Larmor frequency for 3 Tesla MR H+ imaging) and induced in the head model with the DBS implant. The electric field and the SAR distribution were computed using commercially available software (XFDTD v. 7, Remcom Inc., State College, PA) based on the FDTD algorithm [34, 35]. Following the geometrical modeling, two different simulations were performed: (i) using the original mRes model and a 1 mm^3^ uniform electrical grid, and (ii) using the MS model and a finer electrical grid (200 *μ*m^3^) to parameterize the high resolution region of interest surrounded by coarser grids (1 mm^3^) in the rest of the head. Local (1 mm^3^ and 200 *μ*m^3^, resp.) SAR, SAR averaged over 1 g (SAR_1 g_), and 10 g (SAR_10 g_) of tissue were computed. A third simulation was performed using the original mRes model without the DBS implant and used as reference for normalization purposes. The three simulations were normalized to give a maximum whole-head SAR (SAR_w_) for the no-implant case equal to 3.2 W/kg [14]. The total numbers of Yee cells for the grid including the head model with the implant and the coil were 27,638,596 for the mRes model and 104,531,438 for the MS model; the total volume, including the free space around the coil, was 870 × 870 × 894.41 mm^3^. Seven perfectly matching layers were used for boundary conditions in all the models [36]. The timesteps used to ensure FDTD Courant-Friedrich-Levy stability—proportional to the smallest cell size—were 1.07 ps for the mRes model and 0.26 ps for the MS model [34]. The computational time needed to reach a convergence of −40 dB was 98 minutes on a C2070 graphics processing unit (GPU) (Nvidia, Santa Clara, CA, USA) with 6 GB graphics memory for the mRes model and 3 days, 2 hours, and 45 minutes on six C2070 GPUs for the MS model. 

## 3. Results

### 3.1. Anatomical Modeling

**Figure 4:**
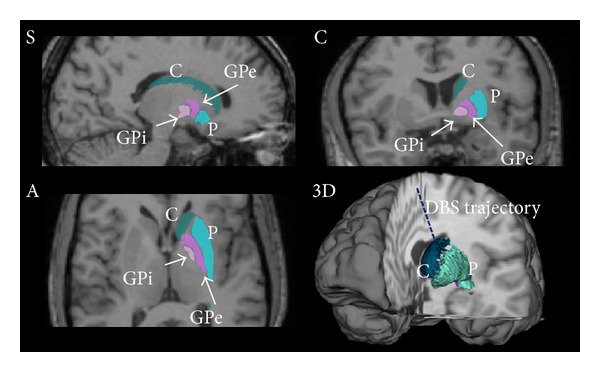
Segmentation obtained via label propagation on the sagittal (S), coronal (C), axial (A), and three-dimensional (3D) views of the 1 mm^3^ resolution MRIs.

Table 1 shows the distances between the centroids (Dc) of the algorithmically and manually segmented structures (denoted as GT) for each structure (caudates, putamens, and GP) and for each structure-specific transformation, namely, the affine (AFF) and the non-rigid (NR) at 20 mm^3^, 10 mm^3^, 5 mm^3^, 3 mm^3^, 1 mm^3^, and 0.5 mm^3^. A resolution of 3 mm^3^ for the final registration grid was chosen. For this resolution, the overlap between the segmented regions and their corresponding GT was assessed using also two volumetric metrics: PM and *P*+. At this resolution level, matching qualities of 86% versus 88% (PM versus *P*+) for the caudate, 96% versus 89% for the putamen, and 71% versus 85% for the GP were found. The accuracies of the final transformations were given by RMSEs of 520 *μ*m, 422 *μ*m, and 479 *μ*m for the caudate, the putamen, and the GP, respectively.  Figure 4 shows the results of the segmentation of the 1 mm^3^ MRIs, the reconstructed 3D model of the structures inside the brain, and the trajectory of the insertion of the electrode. 

### 3.2. Electric Modeling

**Figure 5:**
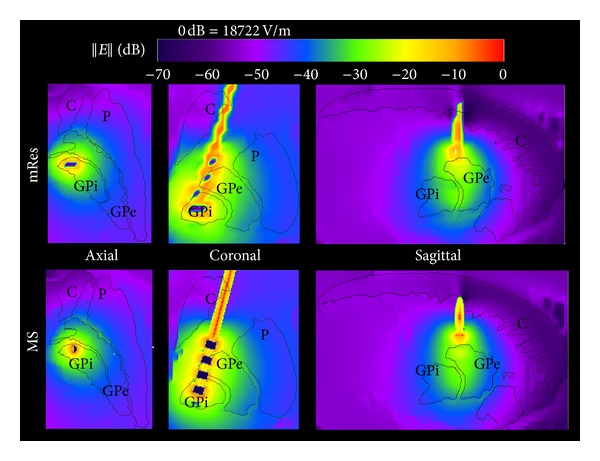
Axial, coronal, and sagittal mappings of the magnitude of the electric field ||E|| around the electrode for the 1 mm^3^ (mRes) and multi-scale (MS) models.

**Figure 6:**
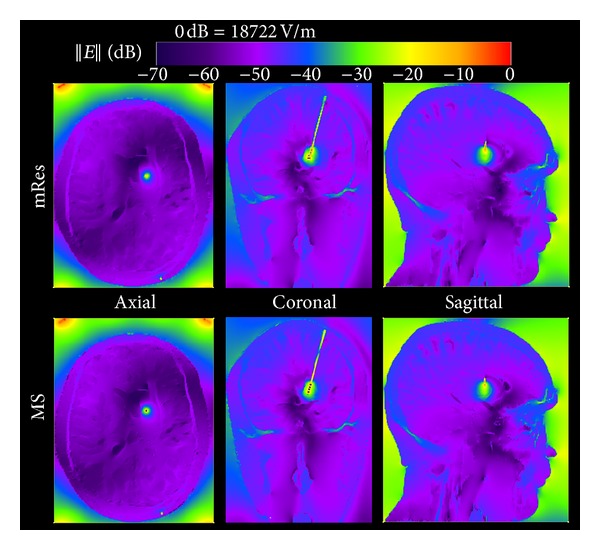
1 mm^3^ (mRes) and multi-scale (MS) whole-head mapping of the electric field along the axial, coronal, and sagittal slices where the maximum electric field was observed.

**Figure 7:**
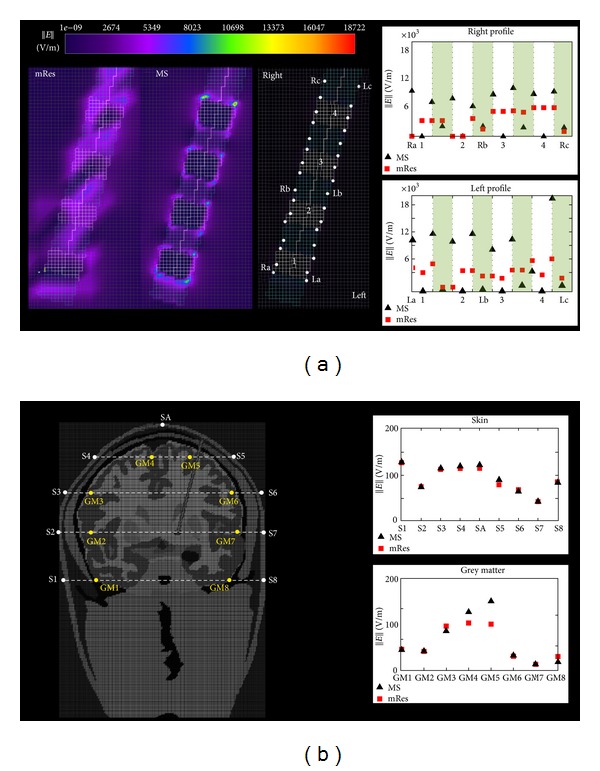
(a) Coronal map of the electric field in proximity of the electrode and difference of the electric field ||E|| between 1 mm^3^ (mRes) and multi-scale (MS) along the right (and left) profile of the electrode from point Ra (La) to Rc (Lc) through point Rb (Lb) (radiological convention, i.e., left is right). Coronal views are shown since the distal part of the DBS is contained in one single coronal plane. (b) Global difference of the electric field ||E|| between mRes and MS configurations in specific positions along two different layers: the grey matter (GM) and the skin (S) layer.

**Figure 8:**
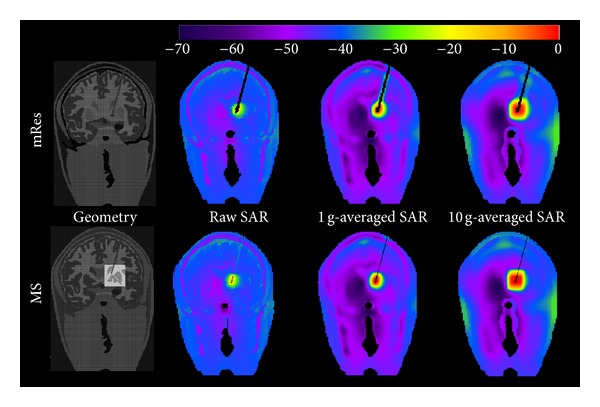
From left to right: anatomical model, raw SAR (0 dB = 43915.78 W/kg), SAR_1 g_ (0 dB = 362.79 W/kg), and SAR_10 g_ (0 dB = 57.90 W/kg) distributions along the coronal slice where the peak is located for the mRes (top) and the MS model (bottom).

Figures 5 and 6 show for both the mRes (*top*) and MS models (*bottom*) the local and global maps of the electric field along the axial, coronal, and sagittal slices. Overall, improvements in spatial resolution affected the electric field computation in the volume surrounding the electrode (Figure 5), while no noticeable differences were noticed in the rest of the head (Figure 6).  Figure 7 quantifies the difference of the electric field calculated using multi-scale and mRes uniform resolution, (i) locally along the right and left profiles of the electrode (top) and (ii) globally along the grey matter (GM) and the skin (S) layers (bottom). The peak of the electric field (18.7 kV/m) was located in the left top corner of the electrode 4 for the MS model, as shown in the figure, while it was halved (9.33 kV/m) and shifted 6.4 mm for the mRes uniform model (not shown). Furthermore, the four conducting electrodes of the implant alternating with the insulation were distinguishable on both the coronal local map and left and right profiles of the MS model (see ▲ versus ■) and corresponded to four local minima (i.e., electric field is equal to zero) alternating with eight local maxima located at the interface electrode/insulation. The difference was 4.14 ± 2.8 kV/m in the profiles of the electrode, 9.34 ± 12.9 V/m in the grey matter layer, and 3.59 ± 3.5 V/m in the skin layer. The SAR_w_ displayed a small change between the MS and the mRes models (3.12 W/kg versus 3.07 W/kg), as expected given the local nature of the antenna effect of the lead. Conversely, (Figure 8) simulations showed a much higher unaveraged SAR values (43.9 kW/kg) computed with basal ganglia modeled using 200 *μ*m^3^ resolution compared to 7 kW/kg obtained with the mRes model. However, when averaged SAR was used, the averaging yielded a greater spatial smoothing effect in the volume surrounding the electrode, with a peak SAR_10 g_ of 57.9 W/kg versus 57.2 W/kg and peak SAR_1 g_ of 362 W/kg versus 317 W/kg for the MS and mRes uniform models, respectively. As shown in Figure 8, the SAR was not computed in the space occupied by the DBS itself.

## 4. Discussion

The objective of this study was to investigate the effect of spatial resolution of the numerical modeling on the calculation of the RF-induced electric field surrounding a DBS implant simulating a patient undergoing MRI. When a numerical model includes objects with fine features, such as implants, the dimensions of the smallest object dictate the maximum cell size of the geometrical mesh, and the computational cost to model an entire head and the coil increases accordingly [9]. Therefore, it is important to assess whether or not an increased spatial resolution is necessary for a precise prediction of the electric field.

A large number of numerical models have been proposed to compute electromagnetic field in the head and the body when internal [37–46] or external [24, 47–58] EM sources are applied. When whole-head measurements are needed, such as in RF absorption during MRI, the domain size is extremely large and the computing time is optimized at the expense of the spatial resolution and the anatomical detail. Conversely, when the local distribution of the electric field surrounding an implant has to be investigated, it is impractical to reduce the size of the mesh to the smallest length scale, and the models are limited to the implant without taking into account the surrounding anatomical structures [20, 33]. Elwassif et al. recently proposed and validated the first finite element method (FEM) model simulating a detailed DBS lead architecture [20]. The experimental validation showed that increased model precision allowed for increased accuracy in estimation of RF-induced heating surrounding the electrode. In that study, the brain was modeled without anatomical information as a cylinder of saline solution. We sought to examine if the precision of the surrounding anatomical structures could affect the electric field computation. To address this question, a multi-scale (MS) geometrical model with both milli- and micro-metric resolutions was constructed to calculate a precise solution of the electric field over the entire head in a reasonable computing time. The milli-resolution (mRes) used in the existing head model [21] allowed precise modeling and visualization of the different anatomical structures of the human head. Micro-resolution (*μ*Res) was crucial to delineate the target, namely, the GPi, for geometrical modeling of the electrode and for its positioning inside the target. 

Previous studies showed that high resolution obtained using high field MRI scanners significantly improved delineation of deep brain structures [22, 23]. We developed a micro-resolution model (atlas) using a T2* MRI dataset of an ex vivo brain hemisphere acquired with a 7 T scanner and an optimized 30-channel receive-only array [25]. The details of the GPi and of the surrounding nuclei—discernible on the ex vivo dataset—were then propagated on the mRes head model using an atlas-based segmentation procedure [59–66]. The enhanced contrast and resolution of the 7 T atlas allowed discernment of the white matter between the GPe and the GPi (i.e., the internal medullary lamina) and thus separating the globus pallidus in its external and internal part, this latter being the target of interest for DBS. This discrimination could not be performed in the previously developed head model because of the limited contrast and resolution of 1 mm^3^ original MRI images. Furthermore, an ex vivo brain dataset was used since a 7 T direct acquisition of in vivo 200 *μ*m^3^ resolution MRIs on a patient with DBS would raise safety concerns and result in long acquisition times with potential motion artifacts. Finally, compared with the previous model [11], micro-resolution also allowed for improved modeling of the electrode, namely, a four-contact DBS lead similar to commercially available models [20]. This resulted in substantial changes in peak SAR near the electrode (123.5 kW/kg for unaveraged SAR and 120 W/kg for SAR_10 g_ [11] versus 7 kW/kg and 57.2 W/kg, respectively of the presented mRes model).

The atlas-based segmentation was performed using a surface-based approach, because the intensity-based registration failed with our original MRI datasets given the different tissue types (i.e., ex vivo versus in vivo) and resolution (i.e., micro- versus milli-resolution). The non-rigid registration was performed for each pair of homologous structures using a standard surface-based registration algorithm, namely, the iterative closest point [26]. The algorithm was performed in a hierarchical fashion from coarse-to-fine resolution in order to achieve a smooth and gradual matching between the structures with low global distortion. ICP superimposes two homologous structures by manipulating a free form deformations (FFDs) control points mesh that parameterizes one of the structures, such that the points of that structure are moved to their closest points on the corresponding structure, reducing the registration to a scattered data interpolation problem. B-splines were used as data interpolation functions because of their local support and allowed for modeling of very complex and localized deformations. While a low resolution FFDs mesh results in a rough registration, a large number of parameter increases the computational cost of the algorithm and may cause local oscillations in the deformation. The non-rigid registration (NR) improved the quality of the segmentation as compared to affine registration (AFF) and, accordingly, with the increase in the resolution of the FFD control point mesh. However, no performance improvement was globally achieved beyond a 3 mm^3^ grid. Furthermore, the volumes of the final structures were comparable with those reported by the previous study of Jovicich and colleagues [67]: left caudate = 3488 mm^3^ (versus 3315 ± 479 mm^3^), putamen = 4978 mm^3^ (versus 4654 ± 848 mm^3^), and globus pallidum = 1938 mm^3^ (versus 1585 ± 218 mm^3^).

The MS geometrical and electrical modeling resulted in increased detail in the calculated local field surrounding the electrode contacts. The MS model allowed clear discernment of the four implant electrodes with a resulting null electric field, as expected given the conductivity of the electrode (Figure 7 top). This detail was not shown by the mRes model because of staircasing limitations. Small differences between the multi-scale and mRes uniform configurations were globally observed when we compared the electric field in specific positions along the grey matter (GM) and skin (S) layers. Only two points in the grey matter, GM4 and GM5, had a difference bigger than 20%. The difference in GM5 is likely due to its vicinity to the DBS: the different resolution of the model determined a difference in the calculated electric field along and near the DBS implant, which includes the point represented by GM5, as well as near interfaces of high electrical discontinuity, such as CSF/Dura/Gray matter, as shown by the results of point GM4. Additionally, the spatial peak SAR calculation was very sensitive to spatial resolution used for the geometric and electric modeling. When the resolution decreases, due to staircasing [68, 69], small structures may be deformed or lost, symmetries may be disrupted, and the three-dimensional spatial consistency of elongated structures may be affected. Therefore, high spatial peak SAR values predicted using finer anatomical models may not be detected using millimetric resolution. Furthermore, the SAR was zero in the space occupied by the electrode. Therefore, the space occupied by the DBS and the relative nonzero SAR values are overestimated when using 1 mm^3^ resolution. Regarding the averaged SAR, smaller differences were observed between mRes and MS models when calculating 1 g-averaged peak SAR (14%), 10 g-averaged peak SAR (1.2%), or whole-head-averaged SAR (16%). 

*Limitations of the Work.* The segmentation is based on the morphing and propagation of the basal ganglia labels from an ex vivo atlas to a head model from a different subject and may result in errors due to anatomical variability. The values of conductivity and permittivity used in the study were found in the literature from in vitro or postmortem measurements in animals [32, 70] and may differ from electrical properties in vivo for human subjects. Furthermore, the dielectric properties of the head model were considered isotropic and did not take into account the white matter fiber direction, which can be considered in a future investigation by including information from diffusion tensor imaging [71]. As in the previous model, our study was based on a generic model of the coil and the truncation of the head model at the neck did not allow realistic modeling of the full length for the DBS implant, usually connected to a stimulator placed on the clavicle. This study showed the computed electric field and SAR during MRI in tissues surrounding a DBS implant and did not include information about temperature, which depends on SAR as well as thermal properties of tissues and thermoregulatory mechanisms of the body [72]. A thermal analysis was beyond the scope of this work, which focused on quantifying the influence of the spatial resolution on the modeling of the electric field and inform decision-making with respect to low versus high resolution modeling. Evaluation of RF-induced temperature changes and thermal damage at high resolution may be considered in a future investigation. 

## 5. Conclusions

The study investigated the effect of the spatial resolution on the calculation of the electric field and SAR around a medical device implanted in a patient undergoing MRI. The method was applied for the analysis of a specific case study, namely, the electric field induced by a 3 T MRI RF birdcage coil along a deep brain stimulator implant and the surrounding brain tissues. An atlas-based segmentation procedure was here used to outline the GPi on a preexisting 1 mm^3^ model and to accurately model the electrode inside the basal ganglia. The highest effect of the high-resolution model was limited to the local electric field and SAR and smaller differences were observed between mRes and multi-scale models when calculating 1 g-averaged peak SAR (14%), 10 g-averaged peak SAR (1.2%), or whole-head-averaged SAR (16%). The method and electromagnetic model herein presented can be used as the foundation for evaluation of RF-induced heating in patients with implanted medical devices. 

## Figures and Tables

**Table 1:** Distances in mm between the centroids (Dc) of each structure (caudates, putamina, and GPs) and each structure-specific transformation, namely, the affine (AFF) to the non-rigid (NR) at 20 mm, 10 mm, 5 mm, 3 mm, 1 mm, and 0.5 mm.

	Dc_Caudates_	Dc_Putamens_	Dc_GPs_
	(mm)	(mm)	(mm)
AFF	3.37	1.26	1.56
NR20	1.64	0.35	0.99
NR10	1.46	0.32	0.82
NR5	1.23	0.23	0.50
NR3	0.98	0.03	0.47
NR1	0.97	0.03	0.49
NR0.5	0.93	0.04	0.46
